# Femtosecond laser-assisted half top hat penetrating keratoplasty for keratoglobus

**DOI:** 10.1016/j.ajoc.2025.102406

**Published:** 2025-07-28

**Authors:** Dahlia Palevski, Noa Reinhertz Marom, Eitan Livny, Yoav Nahum, Samuel Levinger, Irit Bahar

**Affiliations:** a Department of Ophthalmology, Rabin Medical Center, Petach Tikva, Israel; b Gray Faculty of Medical and Health Sciences, Tel Aviv University, Tel Aviv, Israel; c Enaim Laser Center, Jerusalem, Israel

**Keywords:** Keratoglobus, Top hat configuration, Femtosecond laser, Penetrating keratoplasty

## Abstract

**Purpose:**

To describe a modified technique utilizing femtosecond laser-assisted penetrating keratoplasty for the surgical management of keratoglobus with an extremely thin peripheral cornea.

**Methods:**

Two eyes of two patients with advanced keratoglobus underwent a modified, donor-only, top hat keratoplasty. Femtosecond laser-assisted top hat configuration was exclusively performed on the donor cornea (half top hat) and sutured under the recipient's cornea which underwent a regular full thickness trephination. Patients were followed up for 12–24 months after intervention. Visual acuity and anatomical results (measured using corneal tomography and anterior segment OCT) were evaluated after this intervention.

**Results:**

Post-operatively, best corrected visual acuity improved from counting fingers in the first patient and 20/720 in the second to 20/30 in both. Keratometry values markedly decreased, and peripheral thickness was augmented in both cases. No complications during the follow-up period were noted.

**Conclusions:**

Top hat configuration keratoplasty on donor cornea alone may be a unique surgical solution enabling the augmentation of the peripheral cornea in keratoglobus eyes, providing good visual outcomes as well as structural integrity.

## Highlights

• Corneal transplantation in advanced keratoglobus remains a surgical challenge.
• Donor only top hat keratoplasty– effective in keratoglobus with good visual outcomes.
• Donor only top hat configuration - can be applied in other ectatic corneal settings.

## 1 Introduction

Keratoglobus is a rare, non-inflammatory corneal ectasia characterized by generalized thinning and globular protrusion of the cornea. Surgical management of keratoglobus, particularly in cases requiring corneal transplantation, poses significant challenges for which there is no gold standard surgical procedure.^1^, ^2^, ^3^ When considering corneal transplantation for these patients, the surgeon is faced with the technical challenge of suturing a normal thickness graft to an extremely thin recipient peripheral cornea. This graft-host mismatch leads to graft instability and irregular astigmatism, precluding good visual and anatomical outcomes. To address this difficulty, various surgical techniques have been described by different authors, such as two-step sclerocorneal tectonic keratoplasty followed by PK^4^ and lamellar keratoplasty with a peripheral intralamellar tuck.^5^^,^^6^ Each method has its advantages; however, treatment is still technically challenging and may require multiple surgical steps.

With the advent of femtosecond laser-assisted penetrating keratoplasty (PK), various corneal configurations can be precisely designed to allow for optimal graft-host integrity while avoiding the challenging manual dissection of an already fragile cornea.^7^ Several studies have shown that the femtosecond laser can be used to create a top hat PK configuration, and proved its superior mechanical stability compared with the traditional PK graft.^8^^,^^9^

Here we describe a unique approach utilizing femtosecond laser-assisted top hat configuration solely in the donor cornea (half top hat keratoplasty) as a technique providing both good visual outcomes as well as structural integrity in keratoglobus eyes.

## 2 Methods

Two patients signed a detailed consent form prior to undergoing surgery. The use of patient data and accompanying images was approved by the institutional IRB and conformed to the tenets of the Declaration of Helsinki. Before the procedure, both patients had severe keratoglobus with extreme corneal thinning, steepening, and deterioration of vision which could not be improved by non-surgical methods. Patients underwent the same surgical procedure, i.e. donor-only top hat keratoplasty, by the same surgeon (I.B), and donor corneas were prepared in advance in top hat configuration by the same surgeon (S.L). The preoperative medical history of each patient was documented, including a complete eye examination, best corrected visual acuity, slit-lamp examination, intraocular pressure, and fundoscopy. In addition, corneal tomography (Pentacam®, OCULUS Inc.) and anterior segment OCT (Anterion®, Heidelberg Engineering GmbH) were obtained for each patient. Postoperatively, manifest best corrected visual acuity, refraction, and anatomical outcomes such as central corneal thickness and keratometry values were evaluated.

## 3 Surgical technique

**Fig. 1:**
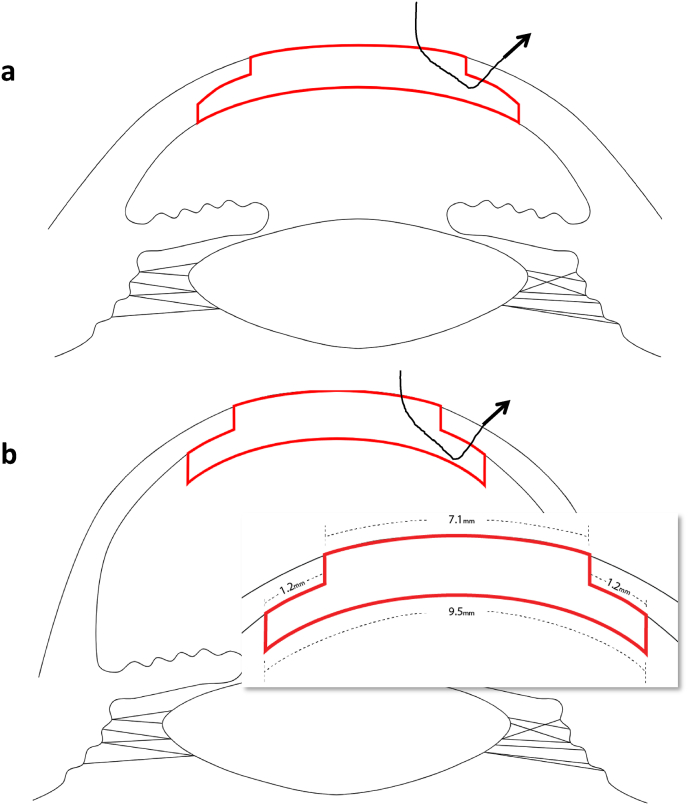
Illustration of the traditional top hat PK (A) vs. donor-only top hat configuration (B) for keratoglobus. Donor graft is sutured under the recipient's full thickness peripheral cornea to allow for mechanically stable host-graft interface.

**Fig. 2:**
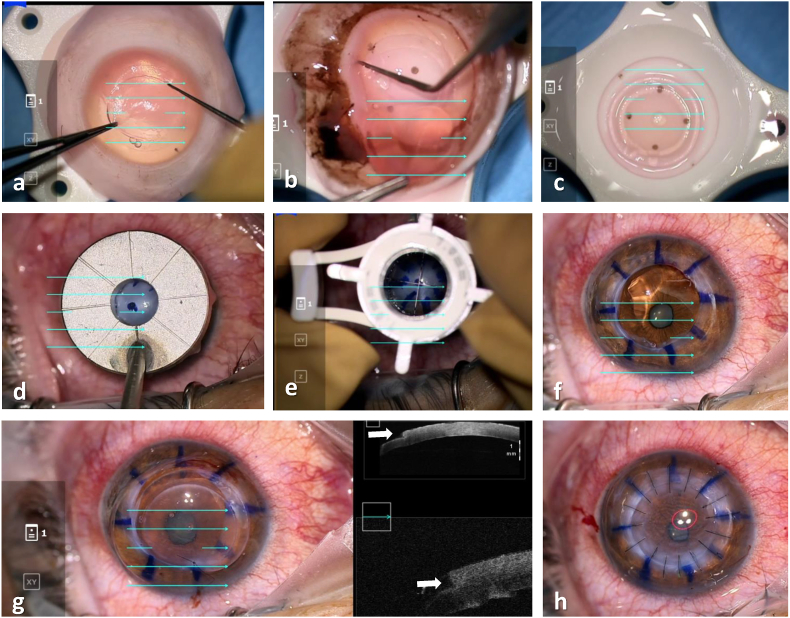
Main surgical steps. A. Anterior scoring of the donor pre-prepared top hat lamellar configuration. B. Posterior scoring and release of donor cornea C. View of the top hat graft with an outer 9.5-mm diameter and an inner, full thickness button of 7.1 mm D. Marking of recipient bed using the 8-zone marker E. Full thickness trephination of recipient bed, 7.0 mm in diameter. F. Filling of the anterior chamber with viscoelastic. G. Suturing of donor graft, each bite passing through the full thickness central button and out through the partial thickness lamellar skirt and host's peripheral cornea. H. View of the cornea after 16 sutures placement under the keratoscope ring.

Femtosecond laser-assisted top hat configuration was performed on donor corneas using the iFS™ 150kHZ advanced femtosecond laser (Johnson & Johnson Surgical Vision Inc.) The laser was programmed to make a 330-μm vertical cut from the endothelium into the stroma (9.5-mm diameter). A horizontal, lamellar, ring cut 1.2 mm in width was completed, followed by a second vertical cut up to the epithelium (7.1 mm diameter). Side-cut angles of 90° relative to the corneal surface were used. The donor button obtained in this way consisted of a central, full-thickness part, 7.1 mm in diameter, surrounded by a peripheral lamellar wing of deep stroma and endothelium that measured approximately 1.2 mm in width (Fig. 1, Fig. 2A -C) to create a 9.5 mm inner diameter. The recipient bed was prepared as in traditional PKP (Fig. 2D–F). A 7.0-mm Hanna suction trephine was used to cut a full-thickness, circular incision in the cornea, centered at the geometric center of the cornea. Excision of the recipient corneal button was completed with curved Castroviejo corneal scissors. The top hat donor button was positioned by sliding the peripheral wing under the trephined recipient bed. 16 interrupted 10-0 nylon sutures were placed, while intraoperative AS-OCT (Zeiss Artevo 800, Carl Zeiss AG, Germany) demonstrated graft-host apposition in top hat configuration (Fig. 2G). Each suture exited the donor button wing and was then passed through the recipient's full thickness lip. An integrated keratoscope ring showed no marked astigmatism at the end of the surgery (Fig. 2H).

Postoperatively, eyes were treated with topical antibiotics qid (Ofloxacin 0.3 %, Oflox; Allergan, Irvine, CA, USA) and steroid eye drops qid (Dexamethasone disodium phosphate 0.1 %, Sterodex, Fischer Pharmaceutical Labs, Tel-Aviv, Israel). Topical antibiotics were discontinued 1 month postoperatively, and topical steroids were gradually tapered down during the first year following surgery.

## 4 Results

### 4.1 Case 1

**Fig. 3:**
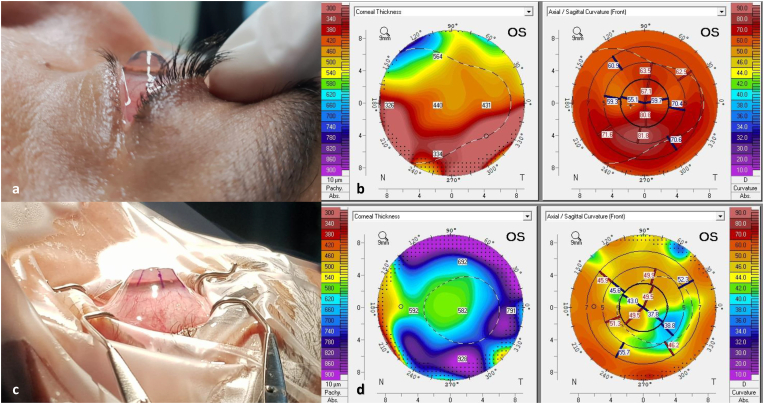
Color photographs and corneal topography before and after donor-only top hat PK. (A) Pre-operative side view shows globular, protruding cornea with a deep anterior chamber. Corresponding tomography before surgery demonstrates thinning and extreme steepness (B). (C) The cornea immediately at the end of surgery demonstrates a flattened surface with markedly reduced depth. (D) Tomography two years after top hat PK shows increased CCT, augmented periphery and normalized anterior curvature. CCT-central corneal thickness; PK- penetrating keratoplasty. (For interpretation of the references to colour in this figure legend, the reader is referred to the Web version of this article.)

A 38-year-old male with a long-standing diagnosis of keratoconus presented with graft failure in his left eye, 20 years after undergoing bilateral penetrating keratoplasty (PKP). Visual acuity in his left eye was counting fingers and slit-lamp examination revealed recurrence of ectatic disease with extreme thinning, bulging of the cornea and deep anterior chamber depth (>5 mm), raising suspicion for keratoglobus as the underlying ectasia. Corneal tomography (Pentacam®, OCULUS inc.) revealed a central corneal thickness (CCT) of 440μm and 326μm at the periphery, with extreme steepness reaching 80.00D (Fig. 3A and B). The cornea was too steep to allow for a proper scleral contact lens fitting. Half top hat keratoplasty was then performed-the donor cornea alone underwent femtosecond laser-assisted top hat configuration to produce a 9.5 mm partial thickness skirt and a central 7.1 mm full thickness button. The patient's original PK graft, measuring around 6.5 mm, was removed by a 7.0 mm trephination. The top hat graft was then sutured under the host's trephined margin. One week following the operation, the patient's best-corrected visual acuity (BCVA) improved to 20/100. Two years postoperatively, his BCVA improved to 20/30 with a refraction of −12.00D −7.00D X 136°. The graft remained clear and stable without evidence of rejection or recurrence of the ectasia. His latest corneal tomography demonstrated increased CCT, and thicker periphery compared with pre-operative values (Fig. 3C and D).

### 4.2 Case 2

**Fig. 4:**
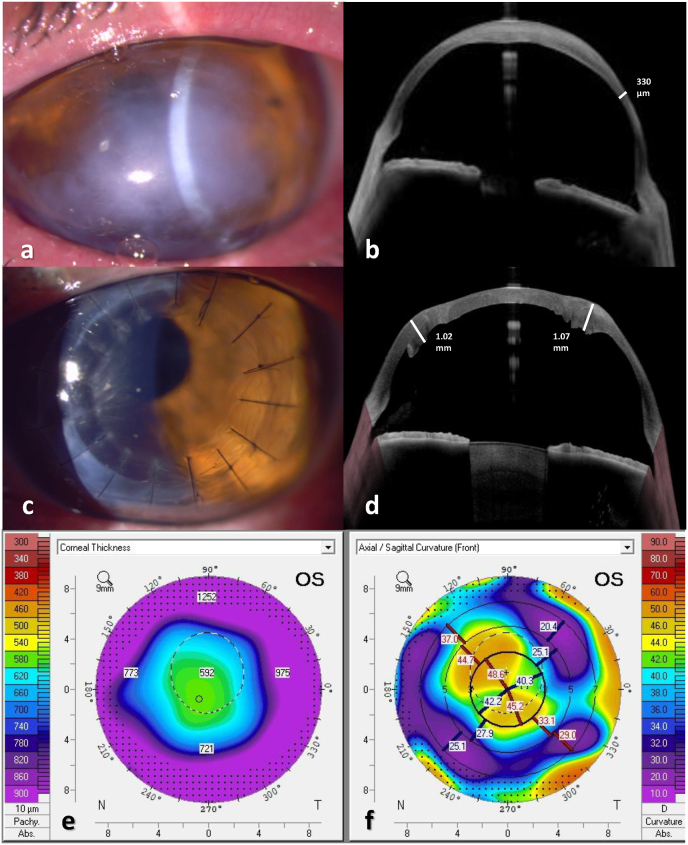
Clinical photographs of patient number 2 before and after donor-only top hat PK. (A) Slit-lamp picture demonstrating peripheral thinning and severe corneal stromal opacification after hydrops, with corresponding AS-OCT (B). (C) At 6-months post-surgery, a clear central full thickness graft with a surrounding haze of lamellar healing is seen. (D) After surgery, CCT measured 529μm and increased thickness (1mm) at graft-host interface was observed (white dashed line). (E) Tomography 12 months after surgery shows augmented peripheral thickness and with-the-rule astigmatism (F). AS-OCT-anterior segment optical coherence tomography. CCT-central corneal thickness.

A 41-year-old male with bilateral keratoglobus and a history of acute hydrops with secondary infectious keratitis in his left eye was referred for corneal transplantation. BCVA was 20/80 in his right eye and 20/720 in the left. Examination revealed an extremely ectatic cornea with stromal edema, scarring and corneal neovascularization in the left eye (Fig. 4A). The corneal tomography in the left eye was unreadable. Anterior segment (AS)- OCT (Anterion®, Heidelberg Engineering GmbH) of the left eye demonstrated a thickened central cornea measuring 920 μm and a thin 330μm cornea peripherally (Fig. 4B). As in the first case, femtosecond laser-assisted preparation of donor cornea was performed prior to surgery to create a top hat configuration with a 9.5-mm diameter and a central 7.1-mm full thickness corneal button. A 7 mm trephination of the recipient's cornea was performed in the left eye, and the customized graft was safely sutured to the host's bed. 12 months post-operatively, BCVA improved to 20/40 in the operated eye, with a refraction of +2.0D −8.25D X32°. The graft was clear, and AS-OCT showed good apposition of graft and host cornea, with a restored peripheral thickness (Fig. 4C and D). Corneal tomography demonstrated a central corneal thickness of 592μm without peripheral thinning, and a regular astigmatism (Fig. 4E and F). Sutures were removed from the steep axis to decrease the remaining astigmatism.

## 5 Discussion

Top hat PK was first described by Busin in 2003.^10^ The advantage of this technique is the combination of both the refractive and structural outcomes of PK with the improved recovery time of lamellar keratoplasty, increased endothelial cell transfer, and faster suture removal. Since then, other studies have shown that top hat PK has several advantages over traditional PK. Manual top hat PK was shown to achieve non-inferior BCVA and refractive results to regular PK, while speeding up suture removal time, allowing for quicker visual rehabilitation.^11^ The increased donor posterior corneal diameter in the top hat configuration also resulted in increased endothelial cell counts one year after surgery. In a following work by Kaiserman et al., half-top-hat PK yielded similar refractive outcomes with faster suture removal compared to regular top hat PK.^12^ Steinert et al. were the first to employ femtosecond laser assisted technology to create top hat lamellar configuration.^13^ Compared to the regular PKP, top hat keratoplasty proved to have superior mechanical stability. In addition, the use of femtosecond laser led to decreased surgery time and proved less demanding than manual dissection top hat configuration. The improved incision stability of femtosecond laser-assisted top hat PKP was further validated by Bahar et al., who have demonstrated that this configuration was the most mechanically stable when compared to other different femtosecond laser assisted configurations: the traditional PK, mushroom, zig zag, and Christmas tree configurations.^7^

In our study, we have demonstrated the feasibility and successful visual outcomes of two patients with severe peripheral corneal thinning consistent with keratoglobus treated with femtosecond laser-assisted half top hat keratoplasty. Femtosecond laser-assisted PK for keratoglobus has been described in one case report to date, where femtosecond laser assisted tuck-in keratoplasty was performed on a 7-year-old patient with severe bilateral keratoglobus. In their report, Alió et al. prepared both the donor and the recipient's cornea in the top hat configuration with good visual outcomes and tectonic stability.^14^ However, dissection of extremely thin corneas poses a significant surgical challenge, and the use of a tuck-in technique may not adequately augment the peripheral thin cornea. Here we describe a modified and relatively simple technique of femtosecond-assisted top hat keratoplasty solely of the donor graft for patients with significant thinning of the peripheral corneas. This augments the peripheral thickness without performing additional manipulation of the keratoglobus cornea such as in tuck-in keratoplasty or lamellar augmentation followed by PK. Using this technique, both our patients achieved good visual outcomes, and no complications were observed during follow-up.

We suggest that this method be considered for any case of full thickness corneal transplantation required in patients with severe mid-peripheral thinning of the cornea (e.g, patients with post graft ectasia). By implementing this approach, one can augment corneal thickness at the graft-host interface, providing proper graft stability.

It is important to note that in our cases, a maximal posterior top hat diameter of 9.5 mm was used due to femtosecond laser limitations. Ideally, increasing donor sizing based on patient's white-to-white measurements in cases with larger eyes such as keratoglobus, would be more effective in providing tectonic stability. However, such customization would require manual preparation of donor grafts, a significantly more challenging, time-consuming approach than our femtosecond laser-assisted technique.

In conclusion, we present a technique of femtosecond laser-assisted half top hat keratoplasty for patients with keratoglobus. This technique is relatively easy to perform and provides good anatomical and visual outcomes.

Further customization of the top hat configuration based on patient corneal anatomy and white-to-white measurements may be necessary to improve tectonic success in the future. A larger series with long follow-up is warranted to confirm the reproducibility and reliability of these findings.

## CRediT authorship contribution statement

**Dahlia Palevski:** Writing – review & editing, Writing – original draft, Data curation. **Noa Reinhertz Marom:** Visualization, Data curation. **Eitan Livny:** Writing – review & editing, Methodology. **Yoav Nahum:** Writing – review & editing, Methodology. **Samuel Levinger:** Writing – review & editing, Methodology. **Irit Bahar:** Writing – review & editing, Methodology, Conceptualization.

## Patient consent

Written consent to publish this case has not been obtained. This report does not contain any personal identifying information.

## Authorship

All authors attest that they meet the current ICMJE criteria for Authorship.

## Funding

No funding or grant support

## Declaration of competing interest

The authors declare that they have no known competing financial interests or personal relationships that could have appeared to influence the work reported in this paper.
